# Detection and risk factor analysis of avian colibacillosis associated with colistin-resistant *Escherichia coli* and *Klebsiella pneumoniae*

**DOI:** 10.3389/fvets.2025.1612542

**Published:** 2025-07-24

**Authors:** Muhammad Adnan Saeed, Haseeb Asif, Syed Ehtisham-ul-Haque, Aman Ullah Khan, Aziz ur Rehman, Aiman Rehman, Muhammad Kamran Rafique, Ishtiaq Ahmed, Muhammad Fiaz Qamar, Herbert Tomaso, Hosny El-Adawy

**Affiliations:** ^1^Department of Pathobiology (Microbiology Section), University of Veterinary and Animal Sciences, Lahore, Pakistan; ^2^Department of Pathobiology (Pathology Section), University of Veterinary and Animal Sciences, Lahore, Pakistan; ^3^Department of Pathobiology (Parasitology Section), University of Veterinary and Animal Sciences, Lahore, Pakistan; ^4^Institute of Bacterial Infections and Zoonoses, Friedrich-Loeffler-Institut, Jena, Germany

**Keywords:** *Escherichia coli*, *Klebsiella pneumoniae*, *mcr*, risk factors, avian colibacillosis

## Abstract

Colibacillosis associated with colistin-resistant avian pathogenic *Escherichia coli* (*E. coli*) poses a threat to both food security and public health. The potential horizontal transmission of mobilized colistin-resistant (*mcr*) genes facilitates the co-emergence of *Klebsiella pneumoniae*. This study aimed to determine the prevalence, molecular detection, analyze the antibiogram and identify associated risk factors for colistin-resistant *E. coli* and *Klebsiella pneumoniae* isolated from broiler chicken in three districts of Punjab province, Pakistan. In total, 230 visceral organ samples were collected from 13 different chicken farms located in Sargodha, Jhang and Toba Tek Singh in Pakistan. Following isolation, the broth microdilution test was used to confirm phenotypic colistin resistance. Polymerase chain reaction was used to detect *mcr-*1 and *mcr-*2 genes associated with colistin resistance. Antimicrobial susceptibility test against 11 antibiotics was performed using the Kirby-Bauer disk diffusion method. Risk factors associated with colistin-resistant bacteria, including host attributes, farm management practices, environmental and agent characteristics, were analyzed. The prevalence of colistin-resistant *E. coli* and *K. pneumoniae* was 24.78% (95% CI, 19.6–30.7%) and 3.04% (95% CI, 1.5–6.1%), respectively. The prevalence of colistin-resistant *E. coli* varied between cities at 42, 23.61 and 5.55% for Jhang, Sargodha and Toba Tek Singh, respectively. The detection frequency of *mcr-*1 gene, 42.1% (24/57), was significantly (*p* < 0.01) higher than that of the *mcr-*2 gene, 14.03% (8/57). Phylogenetic analysis of lipid A phosphoethanolamine transferase sequences revealed greater similarity with *mcr-*1.5 variant. Isolates were found resistant to amoxicillin-clavulanic acid (84.21%), cefotaxime (70.17%), and trimethoprim-sulfamethoxazole (73.68%). The multivariate logistic regression predicted preceding viral infection of the respiratory tract as a significant association (OR = 4.808, *p* < 0.01), whereas daily removal/culling of dead/diseased chicken (OR = 0.308, *p* = 0.01) was a protective factor against the emergence of colistin-resistant strains. These findings indicate that the emergence of colistin-resistant strains deteriorate colibacillosis control efforts in poultry and serves as a possible reservoir for zoonotic infections.

## 1 Introduction

Avian colibacillosis is an infectious disease in chickens caused by avian pathogenic *Escherichia coli* (*E. coli*) (APEC). Colibacillosis is characterized by multisystemic expression of lesions including airsacculitis, perihepatitis, pericarditis, salpingitis, peritonitis, cellulitis, omphalitis and osteoarthritis (1). Colibacillosis poses significant economic losses to the poultry industry worldwide, in terms of mortality, weight loss, decreased egg production, lower hatchability, carcass contamination and costs of prophylaxis and treatment (2). Control of colibacillosis is reliant on strict biosecurity practices, vaccination and antibiotic treatment. Despite standard biosecurity practices adopted on poultry farms, *E. coli* continues to maintain and evolve into diverse strains within the hen house environment via fecal contamination, as the bacterium originates from the avian gut microbiota (3). Due to the diverse plethora of strains and vaccine efficacy only against homologous strains, no single vaccine is effective against all strains; thus, flock-specific autogenous bacterins are often developed for effective prophylaxis (4). Therefore, in most low- and middle-income countries, colibacillosis is treated with the use of antibiotics (2, 5). However, the injudicious use of antibiotics in poultry production and natural evolutionary mechanisms in pathogenic bacteria have resulted in the emergence of multidrug-resistant (MDR) strains of *E. coli* (6, 7). This situation intensifies the challenges of controlling avian colibacillosis.

Antimicrobial resistance (AMR) is a phenomenon of global concern. The use of antimicrobials in food-animal production accounts for 73% of the antimicrobials sold globally (8). Antimicrobials are consumed mainly in terms of achieving productivity goals, maintaining good farm hygiene, and disease control and prevention purposes (8). The importance of antimicrobial resistance genes (ARGs) can never be overemphasized when it comes to the horizontal transfer of genetic determinants of resistance within the populations of pathogenic bacteria, which is critical to the health of both humans and animals (9). Apart from infecting chickens, the resistant clones of *Klebsiella pneumoniae* (*K. pneumoniae*) and *E. coli* have public health significance, as these bacteria can be transmitted from poultry to humans through poultry-origin food products and environmental contamination (10).

Colistin (Polymyxin E) is a cationic polypeptide, broad-spectrum antibiotic, mainly active against Gram-negative bacteria. Despite its nephrotoxicity potential, colistin is considered a last resort antibiotic for treating multidrug-resistant (MDR) Gram-negative bacterial infections due to the unavailability of new antibiotics (11). However, members of the Enterobacteriaceae such as *E. coli*, *Salmonella* spp., and *K. pneumoniae* are becoming increasingly resistant to colistin. One of the earlier known mechanisms of colistin resistance involves chromosomal mutations that activate the two-component regulatory systems PhoP-PhoQ and PmrA-PmrB, causing changes in lipopolysaccharide (LPS) structure, leading to loss of affinity for colistin attachment (12, 13). Further, in 2015, the mechanism of horizontal transmission of colistin resistance was first reported in *E. coli* strains of chicken and porcine origin and *K. pneumoniae* strains of human origin (14). Reportedly, a plasmid harboring the mobilized colistin resistance (*mcr*-1) gene, which encodes the phosphoethanolamine transferase enzyme and adds phosphoethanolamine to lipid A of LPS, causes a reduction in net-negative charge of the outer membrane of Gram-negative bacteria, resulting in loss of colistin affinity (14, 15). More recently, *mcr* gene variants (*mcr-1* to *mcr-10*) have been reported in multiple bacterial species, originating from different sources such as animals, humans, food and the environment (16). These findings highlight the diversity and potential of *mcr* gene to rapidly disseminate colistin resistance.

The medical importance of colistin-resistant bacteria has been well understood. However, little is known about the potential of colistin-resistant avian pathogenic *E. coli*, causing colibacillosis in chickens in low-and middle-income countries like Pakistan. Considering the economic impact and dangers to food security, the present study was conducted to understand the gravity of colibacillosis caused by colistin-resistant *E. coli* in broiler chicken. Therefore, this study aimed to determine the prevalence, molecular characterization, antibiogram, and associated risk factors for colistin-resistant *E. coli* and *K. pneumoniae* in colibacillosis-infected chickens in three districts of Punjab province of Pakistan.

## 2 Materials and methods

### 2.1 Collection of specimens and survey data

In this study, 13 broiler chicken farms located in three cities, Sargodha, Jhang and Toba Tek Singh in Punjab province of Pakistan, were investigated from February 2023 to November 2023. Broiler farms in these specific areas were selected through purposive sampling due to their diagnostic records indicating sporadic colibacillosis outbreaks. A total of 230 multi-organ samples (liver, cecum, heart and lungs) were collected from necropsied broiler chickens with a history of clinical signs and gross lesions (perihepatitis, pericarditis, peritonitis, airsacculitis and omphalitis) associated with colibacillosis (Table 1). All tissue samples were collected in sterile vials, properly labeled and shipped with ice packaging to the Microbiology Research Laboratory, Department of Pathobiology, College of Veterinary and Animal Sciences, Jhang campus, University of Veterinary and Animal Sciences, Lahore, Pakistan. In order to study associated risk factors, information related to farming practices, birds’ health, medication history and farm biosecurity practices was collected from farm managers via a semi-structured questionnaire-based method during interviews, followed by direct observation where possible. All information was gathered and processed in a pre-consented manner and as per the ethical guidelines of the intradepartmental ethical review committee of the University of Veterinary and Animal Sciences, Lahore.

**Table 1:** Distribution of the visceral organ samples collected from necropsied chickens in the study area.

Farm location	No. of farms	Sample type				Total
		Liver	Cecum	Heart	Lungs	
Sargodha	8	36	36	36	36	144
Jhang	3	12	14	12	12	50
Toba Tek Singh	2	8	12	8	8	36
Total	13	56	62	56	56	230

### 2.2 Isolation and identification of *E. coli* and *K. pneumoniae*

For isolation of colistin-resistant bacteria, a previously described method with a few modifications was used (17). Briefly, the collected samples were pre-enriched by inoculation into 10 mL of tryptone soy broth (CM0129, Oxoid, UK) supplemented with 4 μg/mL colistin (Sigma-Aldrich, USA) for selective isolation of colistin-resistant strains and incubated aerobically at 37°C for 24 h. One hundred μL of pre-enriched tryptone soy broth was streaked onto MacConkey agar (Oxoid, Hampshire, UK) supplemented with 4 μg/mL colistin and incubated at 37°C for 24 h. One representative colony from each MacConkey agar plate was subjected to biochemical tests to identify *E. coli* and *K. pneumoniae* species via the analytical profile index (API)-20E kit (bioMérieux, Craponne, France). Aliquots of identified cultures were preserved and stored as 50% glycerol stocks at −21°C until further use.

### 2.3 Phenotypic confirmation of colistin-resistant *E. coli* and *K. pneumoniae*

For phenotypic screening of colistin-resistant isolates, isolated *E. coli* and *K. pneumoniae* were thoroughly swabbed on Mueller-Hinton agar (Oxoid, Hampshire, UK) plates, a colistin disc (10 μg) was applied, and plates were incubated at 37°C for 20 h. All isolates with a diameter of zone of inhibition ≤10 mm were tested further for minimum inhibitory concentration (MIC). MIC was determined via the broth microdilution method by following the guidelines provided by the Clinical Laboratory Standards Institute (CLSI) (18). Briefly, cation-adjusted Mueller-Hinton broth with colistin concentrations (0.25–64 μg/mL) was used, and test cultures with adjusted turbidity equivalent to a 0.5 McFarland standard (1:100 dilution) were used as inoculum. *E. coli* (ATCC 8739) was used as a control organism. Test cultures with MIC value ≥4 μg/mL were considered as colistin-resistant isolates (18).

### 2.4 Antimicrobial susceptibility test

Antimicrobial susceptibility test was performed by using the Kirby-Bauer disk diffusion method. A panel of 11 antibiotic discs including Streptomycin (S-10 μg), Gentamicin (CN-10 μg), Amoxicillin-clavulanic acid (AMC-20/10 μg), Cefotaxime (CTX-30 μg), Ciprofloxacin (CIP-5 μg), Enrofloxacin (ENR-5 μg), Chloramphenicol (C-30 μg), Tetracycline (TE-30 μg), Imipenem (IPM-10 μg), Meropenem (MEM-10 μg) and Trimethoprim-sulfamethoxazole (SXT-1.25/23.75 μg) (Oxoid, Hampshire, UK) was applied. Isolated *E. coli* and *K. pneumoniae* with adjusted turbidity equivalent to 0.5 McFarland standard were swabbed on Mueller-Hinton agar (MHA) plates (Oxoid, Hampshire, UK)., The selected antibiotic discs were applied using sterile forceps and plates were left at room temperature for 30 min, followed by incubation aerobically at 37°C for 24 h. The diameter of zone of inhibition was measured in millimeters and interpreted as resistant, intermediate, or susceptible as per the CLSI criteria (18).

### 2.5 Genomic DNA extraction and molecular detection of mobilized colistin-resistant (*mcr*) genes

Genomic DNA was extracted from 1 mL of an overnight incubated tryptone soy broth culture of colistin-resistant bacteria. DNA was extracted by using the GeneJET genomic DNA Purification kit (K0721, ThermoFisher Scientific, USA) following the manufacturer’s instructions. Polymerase chain reaction (PCR) was conducted using previously reported primer sets to detect mobilized colistin-resistant (*mcr*) genes including *mcr-*1 (Forward: 5′-AGTCCGTTTGTTCTTGTGGC-3′, reverse: 5′-AGATCCTT GGTCTCGGCTTG-3′) and *mcr-2* (Forward: 5′ AGCCGAGTCT AAGGACTTGATGAATTTG-3′, reverse: 5′ GCGGTATCGACAT CATAGTCATCTTG-3′) with generation of PCR product of 320 bp and 576 bp size, respectively (19, 20). Briefly, a total of 50 μL mono-plex PCR reaction mixture was prepared by mixing 25 μL master mix (WizPure™ PCR 2X, W1401-2, Korea), 2 μL each primer (10 μM), 4 μL template DNA and 17 μL of nuclease-free water. Running conditions for amplification in thermal cycler (Biorad, T100, USA) were as follows: Initial denaturation for 15 min at 94°C, 25x (denaturation for 30 s at 94°C, annealing for 90 s at 58°C and extension for 60 s at 72°C) with a final cycle of extension step for 10 min at 72°C. Nuclease-free water was substituted for template DNA in PCR negative controls. DNA from multidrug-resistant (MDR) strains *K. pneumoniae* strain MASJG8 (GenBank: OP744534.1) and *E. coli* strain MASMS_A3 (GenBank: ON736876.1) was used as *mcr-*1 and *mcr-*2 positive controls, respectively. These strains were isolated from our previous studies and maintained at the Microbiology laboratory of the College of Veterinary and Animal Sciences, Jhang. PCR products were electrophoresed in a 1.2% agarose gel at 100 volts for 45 min using the Mupid-One electrophoresis system (Nippon Genetics, Tokyo, Japan). Agarose gel was stained with ethidium bromide (0.5 μg/mL). Gel images were captured and processed using a gel documentation system (Syngene, Cambridge, UK).

### 2.6 Lipid a phosphoethanolamine transferase (*mcr* gene product) phylogenetic analysis

Selected PCR amplicons of *mcr-*1 gene were processed for Sanger sequencing by a commercial service provider, Beijing Genomics Institute (BGI), Shenzhen (518083), China. Sequencing results were submitted to the GenBank database of the National Center for Biotechnology Information (NCBI). A phylogenetic analysis was performed by using corresponding partial protein sequences of lipid A phosphoethanolamine transferase (*mcr-*1 product) obtained in the present study, including GenBank accession numbers WPF45700.1 and WPF45701.1. The comparator and reference sequences included in phylogenetic analysis were obtained from the NCBI public database. This selection was made using the BLAST-p program. The sequences included a reference sequence of *mcr*-1 (HBY7764053.1). The comparator sequences included *mcr*-1.3 (WP077064885.1), *mcr*-1.5 (APM84488.1), *mcr*-1.8 (WP085562407.1), *mcr*-2.1 (WHI19688.1, WHF75690.1), mcr-2.8 (QXM27672.1), *mcr*-3.1 (BBA91300.1, WBW54110.1), *mcr*-4 (QDF67528.1) and *mcr*-4.3 (AYJ09357.1). Class A beta-lactamase CTX-M-1 (WHD27734.1) sequence was added as an outgroup. Whelan and Goldman (WAG) was computed by MEGA software (v 12.0.11) as the best-fit amino acids substitution model based on the lowest BIC (Bayesian information criterion) score (21). A phylogenetic tree was constructed using the maximum likelihood method with bootstraps (500) and the WAG substitution model using MEGA 12 software (22).

### 2.7 Descriptive statistical and associated risk factors analysis

Numerical data was put in Microsoft Excel 365 to calculate percentages and mean values. Regional and comparative prevalence and confidence intervals (CI) of colistin-resistant bacteria were determined using EpiTools (V 2.0) epidemiological calculators (23). Prevalence and mean difference were tested for significance by using one-way analysis of variance (ANOVA) and an independent t-test by considering *p*-values less than 0.05 (*p* < 0.05) as statistically significant. The association of individual risk factors with colistin-resistant bacteria was determined by univariate logistic regression analysis using JASP (Version 0.17.2) (24) to calculate odds ratios (ORs), confidence intervals and *p*-values. Only risk factors with *p* ≤ 0.15 in the univariate logistic regression were selected for inclusion in the final multivariate logistic regression analysis. Selected risk factors were further analyzed via multivariate logistic regression in JASP, wherein both “enter” and automated “backward selection” models were built (24) only factors with *p* < 0.05 at 95% confidence intervals were considered significantly associated with the detection of colistin-resistant bacteria.

## 3 Results

### 3.1 Prevalence of colistin-resistant bacteria

**Figure 1:**
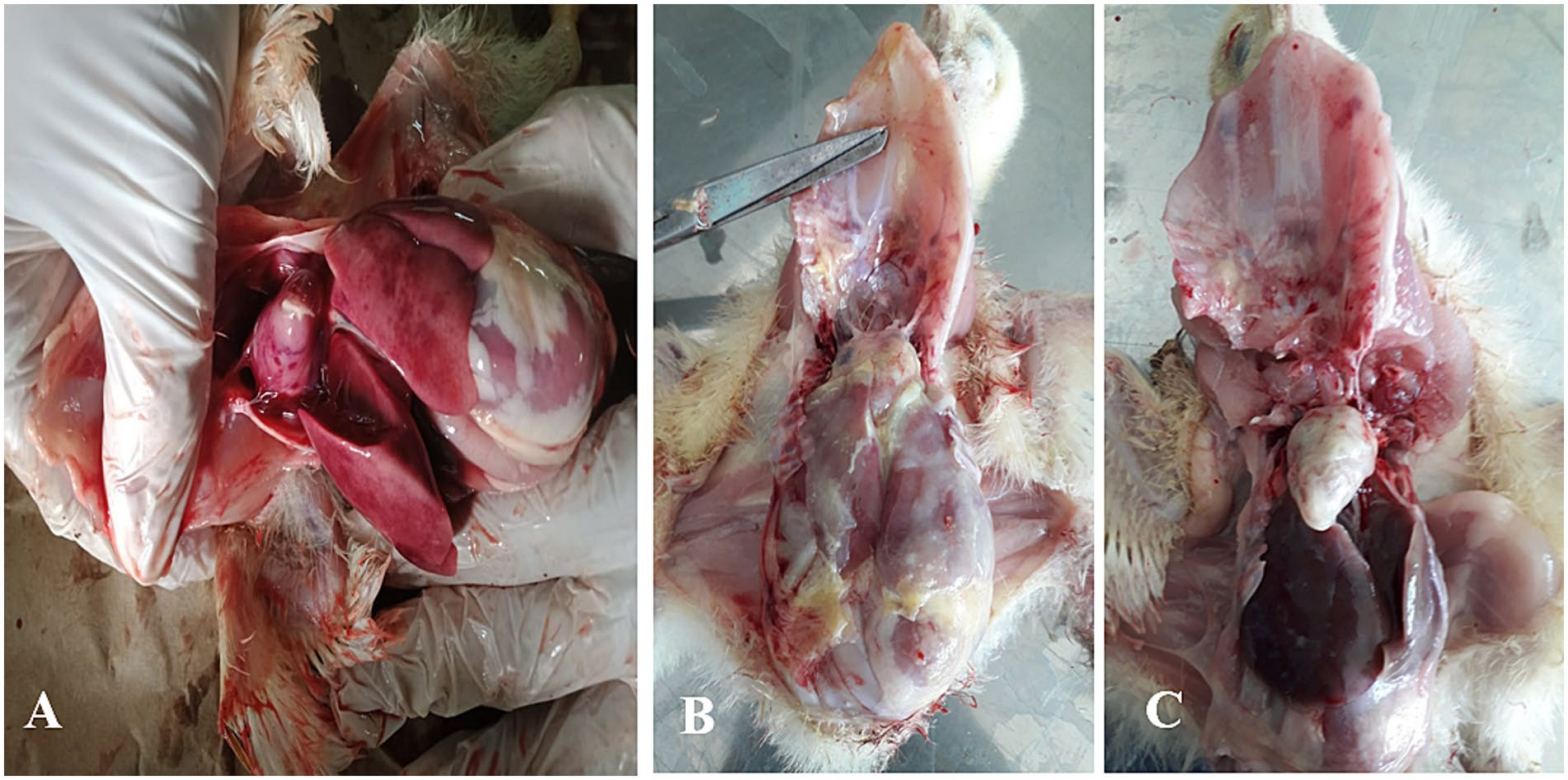
Description of various gross lesions found in necropsy examination of chicken infected with colibacillosis caused by colistin-resistant *E. coli*. **(A)** Multiple necrotic foci are shown on the surface of the heart and liver associated with pericarditis and perihepatitis. **(B)** Fibrous exudate diffusely deposited on the surface of the heart and liver. **(C)** Severe pericarditis marked by creamy fibrous discharge covering the heart surface.

Typical colibacillosis gross lesions, such as severe pericarditis, perihepatitis, airsacculitis and peritonitis, were consistently observed in chickens that died of colistin-resistant strains of avian pathogenic *E. coli* (APEC) (Figure 1). The overall prevalence of colistin-resistant bacteria in collected samples was 27.83% (95% CI, 22.4–33.9%). In a total of 230 collected samples, 57 (24.78%) and 7 (3.04%) isolates were identified as colistin-resistant *E. coli* and *K. pneumonia*, respectively (Table 2). The MIC of colistin for *E. coli* and *K. pneumonia* ranged between 4–16 μg/mL and 4–8 μg/mL, respectively (Supplementary data). The prevalence of colistin-resistant *E. coli* was 24.78% (95% CI, 19.6–30.7%), which was significantly (*p* = 0.01) higher than colistin-resistant *K. pneumoniae*, 3.04% (95% CI, 1.5–6.1%). The prevalence of colistin-resistant *E. coli* varied significantly (*p* = 0.02) between the three studied cities, with the highest in Jhang, 42% (21/50), followed by Sargodha, 23.61% (34/144) and Toba Tek Singh, 5.55% (2/36). Isolation frequency of colistin-resistant *E. coli* was highest in liver samples, 41.07% (23/56), followed by cecum at 33.87% (21/62), heart at 14.28% (8/56) and lungs at 8.93% (5/56).

**Table 2:** Prevalence of colistin-resistant strains of *E. coli* and *K. pneumoniae* recovered from chicken visceral organs in different cities.

Sample collection site	Visceral organs	No. of samples	Colistin-resistant *E. coli n* (%)	Colistin-resistant *K. pneumoniae n* (%)
Sargodha	Liver	36	17 (47.2)	2 (5.55)
	Cecum	36	12 (33.3)	1 (2.77)
	Heart	36	3 (8.3)	0
	Lungs	36	2 (5.55)	0
	Sub-total	144	34 (23.61)	3 (2.08)
Jhang	Liver	12	6 (50)	0
	Cecum	14	8 (57.14)	4 (28.57)
	Heart	12	4 (33.3)	0
	Lungs	12	3 (25)	0
	Sub-total	50	21 (42)	4 (8)
Toba Tek Singh	Liver	8	0	0
	Cecum	12	1 (8.33)	0
	Heart	8	1 (12.25)	0
	Lungs	8	0	0
	Sub-total	36	2 (5.55)	0
Total		230	57 (24.78)	7 (3.04)

### 3.2 Detection and frequency of *mcr*-1 and *mcr*-2 genes in colistin-resistant bacteria

**Figure 2:**
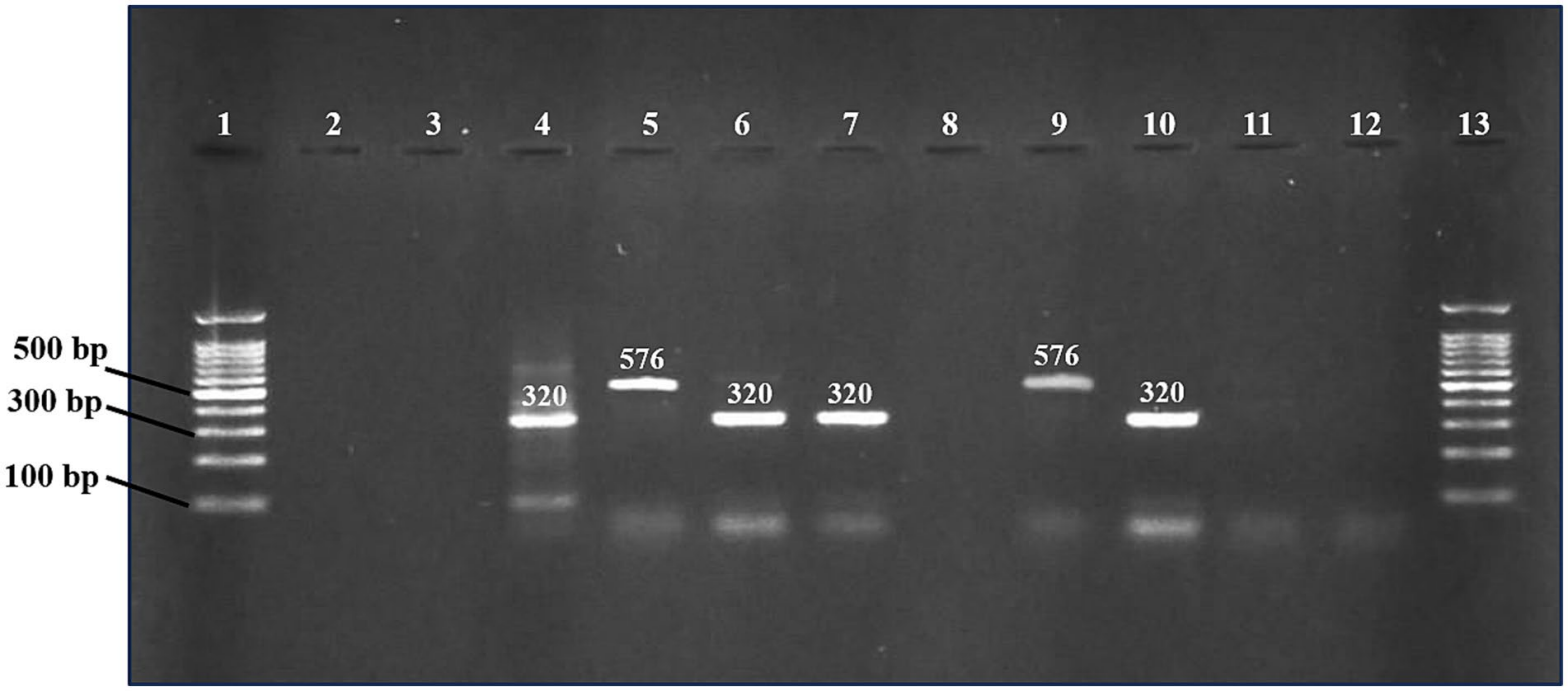
PCR products were detected on a 1.2% agarose gel for *mcr-*1 and *mcr-*2 genes. Lane 1 and 13 (DNA ladder 100 bp, Solis BioDyne, Tartu, Estonia). Lanes 2 and 3 contain negative controls for *mcr-*1 and *mcr-*2, respectively. Lanes 4 and 5 contain positive controls for *mcr-*1 and *mcr-*2, respectively. Lanes 6, 7, and 10 contain positive samples for *mcr-*1 gene (320 bp). Lane 9 contains a positive sample for *mcr-*2 gene (576 bp). Negative samples (lanes 8, 11, and 12).

The *mcr-*1 and *mcr-*2 genes were detected using PCR by amplification of 320 bp and 576 bp products, respectively (Figure 2). In *E. coli* isolates, the detection frequency of *mcr-*1 gene was 42.1% (24/57), which was significantly (*p* < 0.01) higher than that of *mcr-*2 at 14.03% (8/57). However, in 7% (4/57) of *E. coli* isolates, the co-presence of both genes was observed in four isolates (7.01%) (Table 3). In 40.62% (26/64) colistin-resistant isolates, neither *mcr-*1 nor *mcr-*2 genes were detected. The *mcr*-1 gene was identified in two isolates of *K. pneumoniae*, while the *mcr*-2 gene was not present in any of the seven *K. pneumoniae* isolates.

**Table 3:** Frequency distribution of *mcr-*1 and *mcr-*2 genes detected in phenotypically confirmed colistin-resistant *E. coli* and *K. pneumoniae.*

Visceral organs	Bacterial species	No. of colistin-resistant isolates	Genes identified		
			*mcr-*1 *n* (%)	*mcr-*2 *n* (%)	Both (*mcr-*1*, mcr-*2) *n* (%)
Liver	*E. coli*	23	8 (37.78)	2 (8.69)	0
	*K. pneumoniae*	2	1 (50)	0	0
Cecum	*E. coli*	21	11 (52.3)	5 (23.8)	3 (14.2)
	*K. pneumoniae*	5	1 (40)	0	0
Heart	*E. coli*	8	3 (37.95)	1 (12.5)	1 (12.5)
	*K. pneumoniae*	0	0	0	0
Lungs	*E. coli*	5	2 (40)	0	0
	*K. pneumoniae*	0	0	0	0
Total isolates	*E. coli*	57	24 (42.1)	8 (14.03)	4 (7.01)
	*K. pneumoniae*	7	2 (28.5)	0	0

### 3.3 Phylogenetic analysis of lipid a phosphoethanolamine transferase sequences

**Figure 3:**
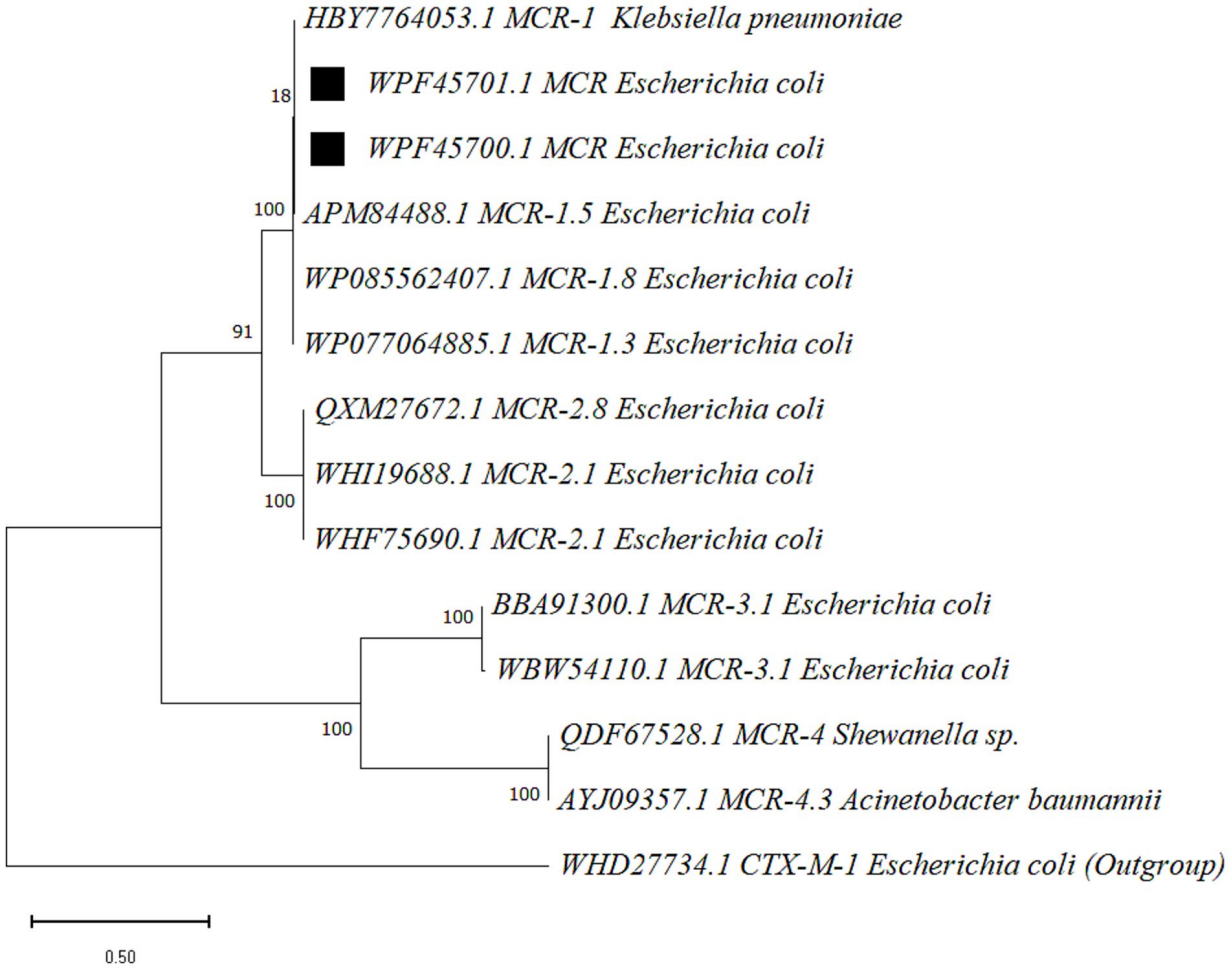
The phylogenetic tree was constructed for *mcr* gene product, lipid A phosphoethanolamine transferase, via MEGA (v 12.0.11) with the maximum likelihood method (bootstraps 500) and the WAG substitution model. Sequences obtained in the present study are marked with black squares. Node labels represent the bootstrap support values (percentage), and the scale bar indicates 0.50 amino acid substitutions per site.

The nucleotide sequences of *mcr* gene products were sequenced and submitted to the National Center for Biotechnology Information (NCBI) with accession numbers OR680710 and OR680711. The corresponding amino acid sequences of *mcr* gene product, lipid A phosphoethanolamine transferase, obtained have accession numbers of WPF45700.1 and WPF45701.1. The maximum likelihood-based phylogenetic tree presented four clades, where *mcr-*1 and *mcr-*2 were found to be the most closely related variants, as compared to *mcr-*3 and *mcr-*4 variants. The sequences of the present study were grouped in *mcr-*1 clade and showed genetic relatedness to *mcr-*1.5 variant (Figure 3).

### 3.4 Antimicrobial susceptibility test

Colistin-resistant isolates of *E. coli* and *K. pneumoniae* were tested for susceptibility against 11 antimicrobial agents belonging to seven different antibiotic classes. Isolates with relatively higher resistance (≥70%) levels were found resistant to amoxicillin-clavulanic acid (84.21%), cefotaxime (70.17%) and trimethoprim-sulfamethoxazole (73.68%). Colistin-resistant *E. coli* showed higher susceptibility to meropenem (98.25%), imipenem (94.74%), streptomycin (78.94%) and ciprofloxacin (73.7%). The susceptibility of *K. pneumoniae* isolates varied from 14.29 to 100% (Table 4).

**Table 4:** Antimicrobial susceptibility profile of colistin-resistant *E. coli* and *K. pneumoniae.*

Antimicrobial class	Antimicrobial agents	*E. coli* isolates (*n* = 57)			*K. pneumoniae* Isolates (*n* = 7)		
		R* *n* (%)	I* *n* (%)	S* *n* (%)	R* *n* (%)	I* *n* (%)	S* *n* (%)
Aminoglycosides	Streptomycin (S-10 μg)	9 (15.8)	3 (5.3)	45 (78.9)	2 (28.6)	1 (14.3)	4 (57.1)
	Gentamicin (CN-10 μg)	18 (31.6)	8 (14)	31 (54.4)	3 (42.9)	1 (14.3)	3 (42.9)
β-lactam	Amoxicillin-clavulanic acid (AMC-20/10 μg)	48 (84.2)	5 (8.8)	4 (7)	6 (85.7)	0	1 (14.3)
	Cefotaxime (CTX-30 μg)	40 (70.2)	11 (19.3)	6 (10.5)	5 (71.4)	1 (14.3)	1 (14.3)
Fluoroquinolones	Ciprofloxacin (CIP-5 μg)	9 (15.8)	6 (10.5)	42 (73.7)	1 (14.3)	2 (28.6)	4 (57.1)
	Enrofloxacin (ENR-5 μg)	27 (47.4)	17 (29.8)	13 (22.8)	1 (14.3)	1 (14.3)	5 (71.4)
Phenicol	Chloramphenicol (C-30 μg)	28 (49.1)	7 (12.3)	22 (38.6)	4 (57.1)	1 (14.3)	2 (28.6)
Tetracyclines	Tetracycline (TE-30 μg)	37 (64.9)	11 (19.3)	9 (15.8)	5 (71.4)	0	2 (28.6)
Carbapenems	Imipenem (IPM-10 μg)	1 (1.8)	2 (3.5)	54 (94.7)	1 (14.3)	0	6 (85.7)
	Meropenem (MEM-10 μg)	1 (1.8)	0	56 (98.3)	0	0	7 (100)
Sulfonamides	Trimethoprim-sulfamethoxazole (SXT-1.25/23.75 μg)	42 (73.7)	3 (5.3)	12 (21.1)	5 (71.4)	1 (14.3)	1 (14.3)

*R, Resistant; I, Intermediate; S, Susceptible.

### 3.5 Risk factors analysis

Risk-associated factors with colistin-resistant bacteria, representing host attributes, farm management practices, environmental factors and agent characteristics, were studied. Initially, univariate logistic regression analysis was performed (Table 5). Univariate analysis found four risk factors including the use of colistin without prescription and/or bacterial sensitivity test (*p* = 0.132), daily removal/culling of dead/diseases chicken (*p* = 0.107), flock history of any preceding viral disease (*p* = 0.138) and bird age (*p* = 0.106) as statistically significant factors with *p* ≤ 0.15. These four risk factors were tested with multivariate logistic regression analysis. The final multivariate logistic regression model was found statistically significant (*χ*^2^ 15.64, *p* < 0.01) with 74% accuracy. Only two risk factors were found statistically significantly (*p* < 0.05) associated with colistin-resistant bacteria (Table 6). These included daily removal/culling of dead/diseased chicken (OR = 0.308, 95% CI = 0.115–0.821, *p* = 0.019) and history of laboratory confirmed viral infection of the respiratory tract (OR = 4.808, 95% CI = 1.961–11.789, *p* < 0.01). Daily culling was found to be protective against the emergence of colistin-resistant bacteria. The risk of the emergence of colistin-resistant bacteria was found to be 4.8 times higher in chickens infected with viral respiratory disease than in those without a history of respiratory viral infection.

**Table 5:** Univariate analysis of the association of potential risk factors with the emergence of colistin-resistant bacteria in broiler chicken farms.

Risk factor	Sub-categories	Colistin-resistant^*^ No. (%)	Colistin-sensitive^*^ No. (%)	Odds ratio	95% Confidence interval		*p*-value
		Total: 64	Total: 166		Lower bound	Upper bound	
Use of colistin without prescription and/or culture sensitivity test	No	22 (9.56)	110 (47.82)	Reference			
	Yes	42 (18.26)	56 (24.34)	0.601	0.310	1.166	0.132^**^
Shed disinfectant type	Fumigation	34 (14.78)	97 (42.17)	Reference			
	liquid disinfectant	30 (13.04)	69 (30)	1.240	0.695	2.215	0.467
Bird’s drinking water disinfection	Yes	29 (12.6)	61 (26.52)	Reference			
	No	35 (15.21)	105 (45.65)	0.701	0.391	1.258	0.234
Rodent and insect control applied	Yes	46 (20)	119 (51.73)	Reference			
	No	18 (7.82)	47 (20.43)	0.991	0.522	1.881	0.977
Farm workers’ biosecurity training and use of gloves and forcovers	Yes	35 (15.21)	90 (39.13)	Reference			
	No	29 (12.6)	76 (33.04)	0.981	0.550	1.751	0.949
Availability of hand washing facility at the farm	Yes	37 (16.08)	80 (34.78)	Reference			
	No	27 (11.73)	86 (37.39)	0.679	0.379	1.215	0.192
Daily removal/culling of dead/diseased chicken^a^	No	33 (14.34)	66 (28.69)	Reference			
	Yes	31 (13.47)	100 (43.4)	0.620	0.347	1.108	0.107^**^
Farming season	Summer	26 (11.3)	75 (32.6)	Reference			
	Winter	38 (16.52)	91 (39.56)	1.205	0.671	2.162	0.533
Flock history of laboratory-confirmed viral infection of the respiratory tract^b^	No	13 (5.65)	50 (21.73)	Reference			
	Yes	51 (22.17)	116 (50.43)	1.691	0.845	3.383	0.138^**^
Bird age	>2 weeks	42 (18.26)	79 (34.3)	Reference			
	≤2 weeks	22 (9.56)	87 (37.8)	0.607	0.332	1.112	0.106^**^
Litter type	Sawdust or wood shavings	36 (15.65)	82 (35.6)	Reference			
	crop remains	28 (12.17)	84 (36.52)	0.759	0.425	1.356	0.352

^*^E. coli and *K. pneumoniae*, ^**^statistically significant (*p* ≤ 0.15).

a Applicability of either one or both.

b Newcastle disease, Infectious bronchitis, Avian Influenza.

**Table 6:** Multivariate analysis of the association of potential risk factors with the emergence of colistin-resistant bacteria in broiler chicken farms.

Risk factor	Sub-categories	Colistin-resistant^*^ No. (%)	Colistin-sensitive^*^ No. (%)	Odds ratio	95% Confidence interval		*p*-value
		Total: 64	Total: 166		Lower bound	Upper bound	
Use of colistin without prescription and/or culture sensitivity test	No	22 (9.56)	110 (47.82)	Reference			
	Yes	42 (18.26)	56 (24.34)	0.804	0.271	2.379	0.693
Daily removal/culling of dead/diseased chicken^a^	No	33 (14.34)	66 (28.69)	Reference			
	Yes	31 (13.47)	100 (43.4)	0.308	0.115	0.821	0.019^**^
History of laboratory-confirmed viral infection of the respiratory tract^b^	No	13 (5.65)	50 (21.73)	Reference			
	Yes	51 (22.17)	116 (50.43)	4.808	1.961	11.789	<0.001^**^
Bird age	>2 weeks	42 (18.26)	79 (34.3)	Reference			
	≤2 weeks	22 (9.56)	87 (37.8)	0.867	0.267	2.812	0.812
(Intercept)				0.260	0.141	0.479	<0.001

^*^E. coli and *K. pneumoniae*, ^**^statistically significant (*p* < 0.05).

a Applicability of either one or both.

b Newcastle disease, Infectious bronchitis, Avian Influenza.

## 4 Discussion

In this study, samples were collected from broiler birds primarily infected naturally with colibacillosis. The postmortem findings revealed organ lesions indicative of pericarditis, perihepatitis, airsacculitis and peritonitis, typically associated with avian colibacillosis. The higher prevalence of *E. coli* in this study is attributed to the fact that avian colibacillosis is caused by avian pathogenic *E. coli* (APEC). However, the detection of colistin-resistant *K. pneumoniae* in this study highlights the possibility of the emergence of secondary pathogens that become active following a primary respiratory tract infection caused by APEC (2). It also indicates the possible transmission of acquired antimicrobial resistance via mobile genetic elements between different pathogenic species of bacteria (25). In the present study, the prevalence of colistin-resistant *E. coli*, 24.78% (95% CI, 19.6–30.7%), was significantly (*p* = 0.01) higher than colistin-resistant *K. pneumoniae*, 3.04% (95% CI, 1.5–6.1%). Isolation frequency of colistin-resistant *E. coli* was highest in liver samples, 41.07% (23/56), followed by cecum at 33.87% (21/62), heart at 14.28% (8/56) and lungs at 8.93% (5/56). During systemic manifestations of colibacillosis infection, APEC colonizes the upper respiratory tract, trachea, and air sacs, followed by colonization of the liver and pericardium via bacteremia (2;19). Bacteremia leads to the multiplication of APEC in organs such as the liver and spleen, where reticuloendothelial tissues are found abundantly (26). Therefore, chicken liver offers a relatively higher isolation sensitivity rate for APEC. The geographical location-based differences in the prevalence of APEC were evident from the present study, interestingly, between the neighboring cities of Toba Tek Singh (5.55%) and Jhang (42%). Factors such as local farming practices, host characteristics, biosecurity, antibiotic usage patterns, vector control and water source have been identified to contribute to the prevalence of APEC at the farm level (27, 28). Similarly, the present study also identified the association of respiratory viral infections and culling management with the prevalence of colistin-resistant *E. coli*. The presence of multidrug-resistant and virulent strains of APEC at farms enhances the risk of transmission to the human population, either through direct contact or the consumption of contaminated chicken products (29, 30).

In this study, the mobilized colistin resistance gene *mcr*-1 was detected in 42.1% of colistin-resistant *E. coli*. The phylogenetic analysis based on two partial *mcr* gene product sequences showed that the amino acid sequences of lipid A phosphoethanolamine transferase were grouped into *mcr-*1 clade next to *mcr-*1.5 variant. These findings are consistent with the findings of previous studies in Pakistan, which reported *mcr-*1 found in bacteria isolated from a wide variety of sources, including colibacillosis-infected chicken (31), poultry farm flies and chicken meat (32). Relatively lower prevalence of colistin-resistant *E. coli*, 18.95 and 7% was reported in Pakistan from healthy poultry birds and livestock in two different studies and only *mcr-*1 gene was detected (33, 34). However, similar to our findings, a recent study targeting samples taken from colibacillosis-infected chicken reported 37% prevalence of avian pathogenic *E. coli* (APEC), wherein 38% isolates were positive for *mcr-*1 gene (31). The *mcr-*1 gene has been reported to be associated with the IncI2 plasmid, which harbors multiple virulence factors and is capable of horizontal gene transfer among carrier *E. coli* (35).

Sampling from internal organs of chicken infected with colibacillosis, as compared to cloacal swabs or fecal samples, allows for isolation of genetically diverse extraintestinal pathogenic strains (36). Hence, depending on the sampling origin, more diverse resistant strains with high sensitivity of isolation can be obtained from diseased chickens as compared to healthy chickens (37).

The *mcr-*2 gene was detected in 14.03% (8/57) of colistin-resistant *E. coli* in the present study; however, to the best of the authors’ knowledge, there is no report of *mcr-*2 gene detected in colibacillosis-infected broilers in Pakistan previously. The *mcr-*2 gene was first reported in *E. coli* isolates associated with diarrhea in calves and piglets in Belgium (38). The *mcr-*2 gene harboring plasmid IncX4 is capable of as high as 10^2^–10^5^-fold transfer frequencies as compared to the epidemic IncFII plasmid (38). These findings explain the rapid horizontal transmission potential of the *mcr*-2 genes in *E. coli*. In China, the *mcr-*2 gene was detected in colistin-resistant *E. coli* isolated from pigs (46.82%), chicken (14.90%) and cattle (19.05%) (39). Similarly, the *mcr-*2 gene has been detected in 3.4% of isolates originating from the chicken gut (3). Furthermore, the co-occurrence of both *mcr*-1 and *mcr*-2 genes has also been reported in colistin-resistant isolates from the same source (40). A study from Egypt found the prevalence of *mcr-*2 in bacteria isolated from resident birds, migratory birds, water sources, and humans as 1.4, 3.6, 11.1 and 9.6%, respectively (41). These studies suggest that the *mcr*-2 gene has evolved in bacteria of multiple sources, including farm animals, chicken, wild birds, humans and the environment. We also found the co-presence of *mcr-*1 and *mcr-*2 in 7% of colistin-resistant *E. coli*. In general, the *mcr-*2 allele has been found globally to be low in prevalence, which varies geographically. In Europe, its prevalence varied from 0.15 to 11.4% in *E. coli*; moreover, the co-existence of *mcr-*1 and *mcr-*2 of swine origin *E. coli* was reported in Germany (42). In Bangladesh, the *mcr-*2 prevalence in chicken has been reported as 3.4%, and co-detection with *mcr-*1 as 1.34% (2/149) (40). In this study, the prevalence of colistin-resistant *K. pneumoniae* was determined as 3.04% (7/230), whereas *mcr-*1 gene was detected in 28.5% (2/7) of colistin-resistant isolates. However, all *K. pneumoniae* isolates were negative for *mcr-*2 gene. Similarly, a study from Egypt found the prevalence of *K. pneumoniae* as 9% (9/100) in commercial chicken, and *mcr-*1 was detected in 18.9% samples (43).

In this study, 40.62% (26/64) colistin-resistant isolates possessed neither *mcr-*1 nor *mcr-*2 genes. The diversity of colistin resistance mechanisms explains the absence of *mcr-*1 and *mcr-*2 genes in colistin-resistant bacteria. There are 10 major variants of *mcr* gene (*mcr-*1 to *mcr-*10) along with sub-variants (16). In addition, chromosomal mutations lead to structural modifications of lipid A, including the addition of 4-amino-L-arabinose or cationic phosphoethanolamine (pEtN) to lipid A, which results in a reduction of negative charge on lipid A and halting colistin coupling with lipid A (25). Such changes are also attributed to the mutations in lipid A biosynthesis genes or via overexpression of chromosomally mediated two-component system genes (PmrAB and PhoPQ) (25). Therefore, different colistin-resistant strains may possess one or multiple different underlying mechanisms for such a phenotype.

Colistin-resistant isolates of *E. coli* and *K. pneumoniae* were tested for susceptibility against 11 antibiotics belonging to seven different classes of antibiotics. *E. coli* with relatively higher resistance (≥70%) levels were found resistant to amoxicillin-clavulanic acid (84.21%), cefotaxime (70.17%), and trimethoprim-sulfamethoxazole (73.68%). While most of the isolates remained susceptible to meropenem and Imipenem. Colistin-resistant *E. coli* were found sensitive to meropenem (98.25%), imipenem (94.74%), streptomycin (78.94%) and ciprofloxacin (73.7%). The susceptibility of *K. pneumoniae* isolates varied from 14.29 to 100%. Similar findings of the highest antibiotic resistance for ampicillin (*β*-lactam group) were found from a previous study, which reported carbapenem-resistant *mcr*-positive *E. coli* associated with avian colibacillosis (31). However, the present study reports a higher resistance rate to amoxicillin-clavulanic acid, thus indicating a wide spectrum of resistance via possible production of inhibitor-resistant β-lactamases and extended-spectrum beta-lactamase (ESBL) type enzymes by resistant bacteria (44). Detection of colistin-resistant and β-lactamase-producing *E. coli* has been isolated from chickens infected with colibacillosis in Tunisia (45). These findings explain the possible resistance mechanisms against third-generation cephalosporins such as cefotaxime. The colistin-resistant *E. coli* in poultry may harbor various genetic determinants that allow for the multidrug resistance phenomenon. In a previous study in Bangladesh, various genetic determinants, including *tet*A (for tetracycline), *sul*1 (for sulfonamide), *aad*A1 (for streptomycin), *aac*-3-IV (for gentamicin) and the two genes *cml*A and *cat*A1 (for chloramphenicol), were detected in chicken meat-associated multidrug-resistant *E. coli* (6).

The risk factors associated with the emergence of colistin-resistant *E. coli* and *K. pneumoniae* in broiler chicken flocks were studied. The final multivariate logistic regression analysis identified two risk factors that were statistically associated with colistin-resistant bacteria. The history of laboratory-confirmed preceding viral infection of the respiratory tract (Newcastle disease, Infectious bronchitis, and Avian Influenza) (OR = 4.808, 95% CI = 1.961–11.789, *p* < 0.01) was positively associated with colistin-resistant bacteria. While farm hygienic measures, daily removal/culling of dead/diseased chicken (OR = 0.308, 95% CI = 0.115–0.821, *p* = 0.019) turned out to be protective, they were found to be associated with reduced risk of emergence of the colistin-resistant bacteria. Depending on the farm management practices, geographical location, virulence of *E. coli* strains and immune status of birds, the risk factors associated with avian colibacillosis can vary (26, 33). The use of groundwater as the source of drinking water, failure to disinfect water channels, farms located in close proximity to other farms, distances greater than 20 meters from car parking to shed and presence of wild birds within 50 meters of the shed surrounding area were found associated with carriage of multidrug-resistant avian pathogenic *E. coli* strains (46, 47). However, in our findings, the use of drinking water disinfectant or groundwater as drinking water has not been identified as a statistically significant factor. These findings may indicate the partial contribution of sewerage contamination of groundwater, with variable levels in different geographical locations. However, a recent study from Jordan identified poor farm sanitary conditions and improper use of antibiotics, especially doxycycline, with the emergence of *mcr-*1 colistin-resistant *E. coli* in broilers (48). The timely and effective culling management, which contributes to overall farm hygiene, has been found to have a significant association. Similar to our findings, a previous study demonstrated that the association of preceding respiratory viral diseases caused by infectious bronchitis virus (IBV), Newcastle disease virus (NDV) and avian metapneumovirus (aMPV) aggravated the avian colibacillosis condition caused by *E. coli* by damaging the respiratory mucosa, tracheitis and airsacculitis (49). Preceding viral diseases are thought to enhance host susceptibility to secondary bacterial infection via multiple factors, including immune deficiency and damage to mucosal barriers.

The present study was limited to the detection of *mcr-*1 and *mcr-*2 genes only. To achieve a more comprehensive understanding of the emergence of colistin resistance in the region, future studies should aim to investigate both *mcr*-mediated and non-*mcr*-mediated resistance mechanisms.

## 5 Conclusion

This study contributed to the understanding of the dissemination of colistin-resistant *E. coli* and *K. pneumoniae* in chickens infected with avian colibacillosis in Pakistan. Mobilized colistin resistance gene (*mcr-*1) was identified as a dominant genetic determinant. However, *mcr-*2 was also detected in *E. coli*. Colistin-resistant bacteria were found resistant to 11 other types of antibiotics, predominantly amoxicillin-clavulanic acid, cefotaxime and trimethoprim-sulfamethoxazole. This study also identified the association of viral respiratory diseases and non-frequent disposal of dead birds and poor culling management as risk factors for avian colibacillosis. The detection of colistin-resistant *E. coli* and *K. pneumoniae* in chickens not only poses a significant threat to food security but also contributes to zoonotic transmission of antibiotic-resistant bacteria. Therefore, efforts must be put in place to conduct genomic epidemiological studies on colistin-resistant bacteria, legislative controls on antibiotic use in animal production and better farm management practices considering associated risk factors in the chicken farming industry in Pakistan.

## Data Availability

The raw data supporting the conclusions of this article will be made available by the authors, without undue reservation.
